# Zymosis

**Published:** 1886-11

**Authors:** James B. Hodgkin

**Affiliations:** Professor Pathology and Therapeutics


### THE DENTAL REGISTER.

VOL. XL.] NOVEMBER, 1886. [No. 11.

### COMMUNICATIONS.

### Zymosis.

(A Lecture before the Class of the Baltimore College of Dental Surgery.)

BY JAMES B. HODGKIN, D.D.S., PROFESSOR PATHOLOGY AND THERAPEUTICS.

I called your attention at our last meeting to the subject of Etiology, or the science of the causation of disease, and on that occasion tried to lay down a few general principles to be kept in mind in studying this subject. We come, this evening, to a branch of this department of pathology which occupies, most probably, a larger place in the mental field of vision of the pathologist than any and all the causes of disease. Indeed, it is looked upon by some as *the* cause, and although I am far from teaching you that disease is one—"a unit,"—yet I should be derelict in my duty as your teacher of pathology not to give this subject a careful hearing.

We are handling, in spite of their high-sounding titles, very simple things, and it is always well, in the outstart to simplify and make plain as we can our subject. Zymosis is the Greek for a *ferment*, and the subject before us is *Ferments and Their Action.* I suppose few of you, as boys, if you lived in the country and had to do with farm life, but have witnessed and taken an active part in making cider. I recall a very early experience in that industry, when it was my function to hitch the old gray horse to the sweep of the apple mill and keep him going on his circular track whilst I kept the mill hopper filled with the apples which were being crushed to a pulp. The almost insipid juice of the sweet cider flowed out under the press and was caught and run into barrels, all under the hot sun of a Virginia August day. Not long did it remain sweet. In a day or so bubbles of a gaseous substance seemed to be formed in the barrel; a foam or froth was forced out of the bung-hole, and a long hollow wheat straw thrust into the depths of the barrel on being sucked by the thirsty boy, brought out, instead of sweet cider, a sparkling, biting fluid, refreshing indeed, and possibly slightly intoxicating, but a very different article from the simple apple-juice lately expressed from the pulp. We said the cider had "worked," and we knew the next step was "hard cider," and still more remotely, vinegar. The philosophy, of course, was unknown to us. That was "fermentation," a chemical process, consisting of, first, a fermentable substance, and, second, a substance capable of setting up the peculiar action we dominate fermentation.

Only last night, as I sat by the fire, the cook brought in the old-fashioned "pan of rolls," wheaten flour made up with water and yeast, and she placed the "batch" before the fire in blissful ignorance of Zymosis, and yet confidently expecting the bread to "rise" against the morning. Here we had for factors, first, starch, and, second, "leaven" or "yeast"—that is a fermentable substance and a substance capable of setting up fermentation.

I take you with me for a little into the laboratory of the wine-maker, old as the deluge and as Noah: possibly taught that Patriarch by Adam himself. I will take a bunch of grapes, and squeezing out the juice into a cup, add a single sporule of yeast. Here are two factors as before, a saccharine liquid and a ferment. The ferment in this case is a yeast-cell, and if we examine this yeast-cell under the microscope we will find it a real plant, a vegetative growth, as truly as a stalk of wheat, or a cabbage. But it is microscopic in size. Its peculiar power, is, first, in its prodigious proliferation or procreative ability, and, second, in its power to cause a subtle change to occur in the grape juice. This is the algebraic proposition. Grape juice plus yeast, equal carbonic acid and alcohol. So in this way the sweet juice of the grape is rendered an intoxicant, as many have seen to their sorrow.

Bear in mind the fundamental proposition that the yeast must have a suitable field for its culture. It might lodge forever in fats or oils with entirely negative results. But we have to remember also the fact that in the case of the sweet cider no ferment was supplied by us, and yet the fermentation went on.

Whence came the ferment in the cider? Whence come the decomposing and fermentative agents which turn milk sour and decompose flesh, and spoil our fruits, and destroy the most careful work of the housewife? Whence come the ferments which work the thousand daily changes we ascribe to chemical action in organic structures? The answer is that the air is full of minute, organic, vegetative germs, which swarm in countless numbers everywhere, whose tenacity of life is simply wonderful, and whose power of multiplication is so amazing as to be beyond our comprehension. I advise all who can to get Tyndal's "Dust and Disease," in order to study the peculiarities of these disease germs. I think he has proven one thing beyond a doubt, and that is that there is no life without previous life, that there is no such thing as "spontaneous generation," a doctrine which some hold even yet. But in this I think that nature is one, and that if a microscopic plant or animal is capable of evolution out of a condition of things, then the highest order of intelligence may be also. The works of nature are on a wonderfully orderly scale and by very exact methods.

Now I have showed you that a yeast sporule can and does do a certain work when in contact with a certain substance by changing this substance into a compound entirely unlike either the yeast or the fermentable grape juice. And I must add further, that in these processes both yeast and grape juice, as such, disappear and are lost, new compounds taking their places

Now to recapitulate, for we must take nothing for granted as understood; in all cases of fermentation we must have, in order to produce the result, first, a substance capable of being fermented; second, a substance capable of inducing fermentation. The grape-juice, the apple-juice, the wheaten flour represented these examples of the first, and the yeast-cell, introduced by the cook, in small quantity, represented the second. And the result of this fermentation, thus set up, be the product alcohol, vinegar, carbonic acid or what, results in the more or less entire destruction of both the yeast and the fermentable articles ; the alcohol, etc., being made at their expense.

So much for the simple *modus operandi* of the matter of fermentation. And now let us see how we can apply this to the study of the causation of disease—etiology.

There is a disease more or less familiar to dirty people, and unfortunately, not unknown to those who, though not dirty themselves, come in contact with personal uncleanness, called the "itch." Until the advent of the microscope it was classed as a *skin disease*, just as we would class an eczema now-a-days as a blood disease; and you know we are given to saying, when we see unwholesome eruptions on the skin, that the patient's blood is out of order. But when this "itch" was studied under the microscope, strange to say, those who investigated the matter, saw living creatures burrowing under the skin, and producing the sores ; and they found that it was only necessary to apply insecticides to the diseased parts to effect a cure.

We are very apt, in our wisdom, to ascribe local effects to general causes. It is, I think, deemed philosophic. But here I tell you of a disease strictly and exclusively local, a living microscopic thing producing disease. Later on, scientists studied more and more with the microscope and enlarged the field of their observations, studying diseased tissues, anxious to find anything which was to be found, and they found in many diseased tissues peculiar animal or vegetable growths, microscopic, possessed of the peculiarities inherent in such organisms, of immense power of proliferation and multiplication, so that where one may be to-day, millions may be to-morrow. These organisms were found so constantly identified with certain diseases that microscopists got into the habit of designating them by the disease with which they were found associated. Then arose the important question : Do these organisms cause the diseases with which they are found associated, or are they simply incidental ? We have not time to go into this subject, one which might not be settled even by a council of the savants of Europe, but we will look over the case as well as we can in a casual way.

First let us note the amazing difference between what we call an ordinary poisonous substance, as opium, strychnia, arsenic, etc., and what is denominated an *organic* poison, as for instance, the peculiar virus of small-pox, or germ of scarlet fever, or some such disease. You are sufficiently familiar with diseases and remedies to know that drugs have medicinal and officinal quantities, and that it takes, all things being equal, a certain amount of morphia, for example, to give your patient relief from pain, and that, further, a certain dose of this is poisonous in the sense of being dangerous to life. But no one familiar with the effects of morphia would expect the one-thousandth of a grain or the one-hundreth part of a thousandth to produce death in any case. In other words the action of such drugs is specific, and can be depended on within reasonable limits.

But when you take the case of an *organic* poison the amazing difference is seen at once. Take scarlet fever, for instance. A person rubbed all over with the infectious germs might not be any more severely attacked, or be any more likely to succumb to the disease, if he took it, than if a single germ were implanted in his system. And what is true of scarlet fever is as true of small-pox. The handling of a bank note, or a letter which had been in the hands of a small-pox patient might prove as potent to bring on a case of confluent and malignant small-pox as though the unfortunate one had slept in the bed with a man sick of this disease.

*The rapid multiplication*, then, of the original organic poison germs is their peculiar characteristic, each one as potent for mischief as its fellow ; there is no such thing as dilution to innocuousness with the segerms.* Now it cannot be scarlet fever is an " influence," or that small-pox is a " condition of things." Take the tiniest probe and dip it in the small-pox matter and introduce it into your flesh and it is enough. Clearly a germ is planted, and you may as confidently look for its fruit in a similar crop as does the farmer who plants corn or sows wheat.

Now let us suppose—life is full of " supposes " more or less reasonable—let us suppose that in the body of the man who is inoculated with small-pox, a substance, we may say in his skin

* Possibly this statement should be somewhat modified in the light of the experiments of Pasteur on the attenuation of the poison of rabies.

and mucous membrane, capable of acting a part analogous to the grape juice mentioned in the first part of this lecture. Let us assume that the small-pox germ plays the part of the yeast, and the "something" in the man plays the part of the grape juice, and how startling the likeness! The rapid multiplication of the germs, the combination of these with the peculiar tissue in which they thrive; the destruction in the system of the germs, and hence the recovery of the patient, when he does recover; the immunity of the patient afterwards—all these show, first, that there was a specific poison. Second. That it found fruitful or congenial soil. Third. That it acted on and combined with certain tissues or substances in the body. Fourth. That these were either destroyed or so changed as to be incapable of subsequent inoculation. Fifth. That the germs themselves either escaped from the body or were destroyed in it by combination with the matter capable of nourishing them.

We all know how certain diseases seem to have special affinities for certain parts of the body. Typhoid fever, for example, produces ulceration of a part of the bowels. Diptheria localizes itself in the throat, etc. If I had time it would be curious to see how many coincidences occur of which this theory supplies a solution. The more we think about it the more we will see that the theory of Zymosis supplies an explanation of much that is obscure in etiology. That it is perfectly satisfactory is not to be expected; no theory is. But it is strongly in harmony with much that is known. Let us look, for instance, at one fact. The bichloride of mercury is now well known as a "germicide," to use the modern term. Housewives have used it for many years as an effective remedy for bed-bugs in the spring cleaning. These germs were very visible and tangible, but it was later found that this same bichloride of mercury in solution in water, even in the one-thousandth part, would destroy such germs as produced mortification in wounds and angry sores from scratch.

Most of us have had that minor affliction, a "boil." In my youth I was told that my "blood was bad," though why the bad blood took that eruptive form I never could see. But I was often struck with the fact that the boil, in every case at first came invariably with a little pustule, which "mattered" and then the boil proper set in and spread into the tissues. In every case there were two stages of progress. Now, thanks to the microscope, we know that the first stage is the first crop of organisms which hatching out a numerous brood, spread into the adjoining tissues, producing inflammation and its usual sequelæ.

Take another illustration, and it shall be my last, of a tooth with a dead pulp. It is dead, as you discover, by the usual signs, but its owner tells you that it has never given any trouble. You are anxious to improve its appearance, and drill into it to obtain access to the pulp chamber to whiten it. You may not do any more at this sitting than merely to open into the pulp chamber and in a few minutes close up the opening you have made, yet it may be long enough to allow access to the pulp chamber of germs which, setting up fermentation in the already dead tissues in that chamber, may produce intense inflammation and suppuration, with alveolar abscess and no little constitutional disturbance. There seems to be no doubt, in conclusion, that inasmuch as we *know* that certain organisms will produce fermentation, and that certain drugs will kill these organisms, that the study of the future medical scientist will be in this direction.

